# Complex Formation between NheB and NheC Is Necessary to Induce Cytotoxic Activity by the Three-Component *Bacillus cereus* Nhe Enterotoxin

**DOI:** 10.1371/journal.pone.0063104

**Published:** 2013-04-30

**Authors:** Uta Heilkenbrinker, Richard Dietrich, Andrea Didier, Kui Zhu, Toril Lindbäck, Per Einar Granum, Erwin Märtlbauer

**Affiliations:** 1 Department of Veterinary Sciences, Ludwig-Maximilians-Universität München, Oberschleissheim, Germany; 2 Department of Food Safety and Infection Biology, Norwegian School of Veterinary Science, Oslo, Norway; Consejo Superior de Investigaciones Cientificas, Spain

## Abstract

The nonhemolytic enterotoxin (Nhe) is known as a major pathogenicity factor for the diarrheal type of food poisoning caused by *Bacillus cereus.* The Nhe complex consists of NheA, NheB and NheC, all of them required to reach maximum cytotoxicity following a specific binding order on cell membranes. Here we show that complexes, formed between NheB and NheC under natural conditions before targeting the host cells, are essential for toxicity in Vero cells. To enable detection of NheC and its interaction with NheB, monoclonal antibodies against NheC were established and characterized. The antibodies allowed detection of recombinant NheC in a sandwich immunoassay at levels below 10 ng ml^−1^, but no or only minor amounts of NheC were detectable in natural culture supernatants of *B. cereus* strains. When NheB- and NheC-specific monoclonal antibodies were combined in a sandwich immunoassay, complexes between NheB and NheC could be demonstrated. The level of these complexes was directly correlated with the relative concentrations of NheB and NheC. Toxicity, however, showed a bell-shaped dose-response curve with a plateau at ratios of NheB and NheC between 50:1 and 5:1. Both lower and higher ratios between NheB and NheC strongly reduced cytotoxicity. When the ratio approached an equimolar ratio, complex formation reached its maximum resulting in decreased binding of NheB to Vero cells. These data indicate that a defined level of NheB-NheC complexes as well as a sufficient amount of free NheB is necessary for efficient cell binding and toxicity. Altogether, the results of this study provide evidence that the interaction of NheB and NheC is a balanced process, necessary to induce, but also able to limit the toxic action of Nhe.

## Introduction

*Bacillus cereus* is known as a causative agent of two different types of food poisoning (for reviews, see references [1], [2]), which are characterized by either emesis or diarrhea. Whereas the emetic type of food poisoning is caused by a heat-stable cyclic peptide (cereulide, [3]), the diarrheal type has conventionally been related to cytotoxin K, a single protein [4], as well as to two enterotoxin complexes, each consisting of three different exoproteins. Hemolysin BL (Hbl) was first described in 1994 and contains the protein components B (37.5 kDa), L1 (38.2 kDa), and L2 (43.5 kDa) [5], [6]. Shortly after, the nonhemolytic enterotoxin (Nhe) was identified in the cytotoxic *B. cereus* strain NVH 0075/95 lacking Hbl, isolated after a large food-poisoning outbreak in Norway [7]. Sequencing and characterization of the *nhe* genes of *B. cereus* strain NVH 1230/88, responsible for another food-poisoning outbreak in Norway [8], revealed that the genes encoding the three protein components NheA (41.0 kDa), NheB (39.8 kDa) and NheC (36.5 kDa) are transcribed as an operon [9], [10]. Comparison of the individual components of both Nhe and Hbl showed a significant degree of sequence homology within each complex as well as between the Nhe and Hbl proteins [11]. Based on sequence homology to Hbl-B, for which the X-ray crystal structure has been established [12], homology modeling of both NheB and NheC indicated a mainly α-helical structure with a hydrophobic β-tongue and overall strong similarities to ClyA [11], a cytolysin forming α-helical pores [13].

Nhe acts as a pore-forming toxin with a specific binding order of the three components [14], in which the presence of NheC is mandatory in the priming step. NheA is obligatory in the final step and triggers toxicity by a so far unknown mechanism. NheB binds to cell membranes independently of the other components. However, a fully active toxin complex is formed only when NheB is applied together with NheC or after cell priming with NheC. Interaction between NheB and NheC seems to occur in solution before cell binding [10], [14], and the optimum molar ratio between NheA, NheB and NheC is near 10:10:1. If, however, an equimolar concentration of NheC is added to a solution containing NheA and NheB, a complete inhibition of cytotoxic effects will occur [10].

Considering that NheC is able to interact with NheB in solution and that toxic activity of Nhe is inhibited when NheC exceeds a critical “threshold concentration”, we assumed that NheC may form a complex with NheB, which could have an enhancing as well as a limiting effect on the mode of action of Nhe. To prove this hypothesis, we completed the set of antibodies available against Nhe components [15] by preparing monoclonal antibodies against NheC. The results obtained using these new analytical tools provided insight into the complex formation between NheB and NheC, their functional consequences and allowed compiling of a more detailed model for the initial step of pore formation by the nonhemolytic enterotoxin of *B. cereus*.

## Materials and Methods

### Ethics Statement

Immunizations of mice for generating monoclonal antibodies were conducted in compliance with the German Law for Protection of Animals. Study permission was obtained by the Government of Upper Bavaria (permit number 55.2-1-54-2531.6-1-08).

### *B. cereus* Strains, Culture Medium, and Culture Conditions

*B. cereus* reference strains used in this study were NVH 1230/88 (producing Nhe, Hbl and CytK; [8]), NVH 0075/95 (producing Nhe; [7]), MHI 1761 (producing NheB and NheC; [14]) and MHI 1672 (producing NheA and NheB; [14]) as well as the *B. cytotoxicus* NVH 0391/98 (producing cytotoxin K; [4], [16]). For toxin production *B. cereus* was grown in casein hydrolysate glucose yeast (CGY) medium supplemented with 1% glucose and treated as described previously [14]. Wild-type NheB was purified from 5- to 6-h culture supernatants of MHI 1672 by immunoaffinity chromatography (IAC), and purity was documented by sodium dodecyl sulfate-polyacrylamide gel electrophoresis (SDS-PAGE) [15]. The histidine-tagged recombinant NheA (rNheA) [17] was expressed in *E. coli*, grown in Luria-Bertani (LB) medium supplemented with the required antibiotic.

### Expression of Recombinant NheC (rNheC)

A plasmid containing histidine-tagged *nheC* from *B. cereus* strain NVH 1230/88 [10] was used to transform *E. coli* BL21(DE3)pLysS (Novagen, Germany). For toxin production, cells were grown in LB medium supplemented with glucose, ampicillin (50 µg ml^−1^) and chloramphenicol (34 µg ml^−1^) with shaking at 37°C for 17 h. 10% of the overnight culture was transferred to fresh LB medium with the corresponding additives, shaken at 37°C until reaching an OD_600_ of 0.5–0.6 followed by 1 mM IPTG induction for 4 h. Cell pellets were obtained by centrifugation (1.900×g at 4°C for 20 min) and frozen at −20°C overnight. Cell disruption was achieved by lysing the pellet in 8 M urea, 100 mM NaH_2_PO_4_ and 10 mM Tris (adjusted to pH 8 with NaOH as defined in Qiagen Ni-NTA Spin Handbook), followed by sonication for 30 min. Subsequent to centrifugation (1.900×g, 4°C, 15 min) the supernatant was dialyzed against phosphate-buffered saline (PBS) at 4°C for 3 days (three changes of buffer).

### Purification and Quantification of rNheC

Affinity purified anti-peptide antibodies (17 mg), derived from the IgG-fraction of a rabbit antiserum specific for NheC [15], were coupled to 1 g of CNBr-activated Sepharose 4B (GE Healthcare, Germany) according to the manufacturer’s instructions. The resulting gel (3.5 ml) was used in a 5 ml column for the purification of rNheC under the following conditions: (i) storage buffer (PBS, containing 0.1% sodium azide) was replaced with PBS; (ii) 35 ml sample (*E. coli* supernatant dialyzed against PBS) were applied; (iii) column was washed with 20 ml PBS; (iv) bound rNheC was eluted with 16 ml glycine/HCl buffer (pH 2.5); immediately after elution pH was adjusted to 7.0 with 1 M Tris base; (v) column was washed with PBS and stored in storage buffer. The flow rate was constantly kept at 1 ml min^−1^ throughout all steps. Eluted protein was dialyzed against PBS at 4°C for 3 days (three changes of buffer) and rNheC was stored at 4°C.

The purity of the toxin was checked by precasted SDS-PAGE minigels (Phast Gel gradient 10 to 15%) in a Phast System (GE Healthcare, Germany). Protein bands were visualized by SYPRO Ruby protein gel stain (Invitrogen, Germany) according to the manufacturer’s protocol on a Kodak image station (Eastman Kodak, USA). Quantification was carried out by densitometry using bovine serum albumin (BSA) as a reference and TotalLab Nonlinear Dynamics Image Analysis Software (TotalLab, USA) for calculation.

### Production of Monoclonal Antibodies (MAbs)

Immunization of mice, cell fusion experiments, establishment of hybridomas, and antibody purification were done as described by Dietrich et al. [18]. Purified rNheC was used as an immunogen for a group of five 12-week-old female hybrid mice [BALB/c × (NZW × NZB)]. Each mouse received 40 µg of rNheC, dissolved in 20 mM Tris base-HCl buffer (pH 7.4) and emulsified in Freund’s adjuvant. A first booster injection of another 40 µg of rNheC (in PBS) in incomplete Freund’s adjuvant was applied at day 135. Three days before cell fusion, at day 176, a final booster injection of 60 µg rNheC dissolved in PBS followed.

### Indirect EIA

To screen for antibody secreting hybridomas and to determine the relative antibody titers, an indirect EIA system was established [18] by using purified rNheC (1 µg ml^−1^) as coating antigen and a horseradish-conjugated rabbit-anti-mouse antibody (Dako, Germany) at a dilution of 1:2,000 in 1% sodium-caseinate-PBS for detection. This indirect EIA system was also applied for further characterization of monoclonal antibodies reactive with NheC, with some modifications. Plates were coated with serial dilutions of purified rNheC and NheB, supernatants of *E. coli* expressing rNheA, as well as supernatants of *B. cereus* strains NVH 1230/88, NVH 0075/95 and MHI 1672. MAbs against NheC were added at a concentration of 1 µg ml^−1^ PBS and as a control NheC-specific rabbit antiserum [15] at a dilution of 1:1,000 in PBS, which was detected by a swine anti-rabbit antibody (Dako, Germany; diluted 1:2,000 in 1% sodium-caseinate-PBS).

### Cytotoxicity and Neutralization Assay

Cytotoxic activity of purified rNheC in combination with *B. cereus* MHI 1672 supernatant was verified using Vero cells and water-soluble tetrazolium salt (WST-1; Roche Diagnostics, Germany) as described previously [14], [18]. In order to analyze the neutralization capacity of the MAbs against NheC, this Vero cell assay was modified as described for neutralization of NheB-related cytotoxicity [17]. In a simultaneous assay, serial dilutions of *B. cereus* strain NVH 0075/95 alone or serial dilutions of purified rNheC together with a constant amount of MHI 1672 were incubated with 10 µg of the MAbs (1 mg ml^−1^ PBS). In a consecutive assay, serial dilutions of rNheC were placed into the microtiter plates together with 10 µg of the MAbs, followed by a constant amount of MHI 1672 in the second step. In both assays 10 µg of an unrelated MAb (MAb 5B2 against 3-acetyldeoxynivalenol, a *Fusarium* mycotoxin) served as a control.

### Western Immunoblot Analyses

Samples were separated by SDS-PAGE, transferred to a PVDF-membrane (Immobilon-P; Millipore, USA) and blocked with 3% sodium-caseinate-PBS containing 0.025% Tween 20. The membrane was incubated for 1 h with the monoclonal antibodies (purified MAb 2 µg ml^−1^, MAb-supernatants 1:10 in 3% sodium-caseinate-PBS supplemented with 0.025% Tween 20) or with NheC antiserum at a dilution of 1:500 as a positive control. After three steps of washing in PBS containing 0.1% Tween 20, horseradish-conjugated swine anti-rabbit or horse anti-mouse antibodies (Cell Signaling Technology, USA) diluted 1:2,000 were applied for 1 h. The membrane was washed three times in PBS-Tween 20 (0.01%) and twice in PBS. Finally, Super Signal West Femto Maximum Sensitivity Substrate (Pierce, USA) was applied and luminescence was recorded on a Kodak image station (Eastman Kodak, USA).

### Epitope Mapping

Epitope mapping was done by PEPperPRINT GmbH (Heidelberg, Germany), as described by Stadler et al. [19]. In brief, the protein sequence of NheC (derived from *B. cereus* strain NVH 1230/88) was displayed as 13mer overlapping peptides with a shift of one amino acid. C- and N-termini were elongated by neutral GSGSGSGSG linkers. The peptides were spotted on a microarray (PEPperCHIP®, PEPperPRINT GmbH, Germany) and incubated with the four monoclonal antibodies (1 µg ml^−1^) at 4°C overnight. Peptide microarrays were stained with fluorescence-labeled secondary antibodies and read by an Imaging System (Odyssey Imaging, USA).

### Competitive Binding Analyses

Competitive binding of the antibodies was tested according to the method described by Friguet et al. [20] with some modifications. Microtiter plates were coated with NheC-specific rabbit antiserum, blocked and then rNheC was added at a concentration of 12.5 ng ml^−1^ for 1 h. After washing, saturating amounts (500 ng ml^−1^) of the MAbs were added either separately or in combination of two different antibodies. Bound MAbs were detected by a rabbit anti-mouse antibody labeled with horseradish peroxidase (Dako, Germany; diluted 1:2,000 in 1% sodium-caseinate-PBS). Under these conditions, non-competitive binding of an antibody pair results in an additive increase of the assay response and the relative additivity index was calculated for each antibody pair [20].

### Immunofluorescence Microscopy

Vero cells (Bio Whittaker, Belgium) were seeded in 8-well Lab-Tek chamber slides (Nunc, Germany) at a density of 60,000 per well and cultivated at 37°C in an atmosphere of 7% CO_2_ overnight. Cells were incubated with purified rNheC (400 ng ml^−1^ or 1.6 µg ml^−1^, corresponding to 125 and 500 ng per well) or fresh cell-culture medium as negative control for 2 h, washed carefully and subsequently fixed in ice-cold methanol for 10 min. After blocking with 5% inactivated goat serum (MP Biomedicals, Germany) for 1 h, MAbs against NheC were added. MAbs were detected by Alexa Fluor 488-conjugated anti-mouse secondary antibody (Invitrogen, Germany) at a concentration of 15 µg ml^−1^. Goat serum and the MAbs against NheC were diluted in PBS containing 1% bovine serum albumin (BSA). Nuclei were counterstained with DAPI (Prolong Gold antifade reagent with DAPI; Nunc, Germany) and the slides were screened with a BZ-8000 fluorescence microscope (Keyence, Germany). Cells were analyzed by z-series, using full focus and haze reduction. Confocal pictures were taken with a Hitachi HV-C20A (Hitachi, Japan) digital camera connected to a Zeiss LSM 510 microscope (Carl Zeiss GmbH, Germany).

### Flow Cytometry

Vero cells were grown to confluency, trypsinated and adjusted to 1,000,000 cells per ml in EC-buffer (140 mM NaCl, 15 mM HEPES, 1 mM MgCl_2_, 1 mM CaCl_2_, 10 mM glucose pH 7.2). Experimental set-up was as follows: i) Vero cells without treatment; ii) Vero cells incubated with MHI 1761 (natural ratio of NheB : NheC 10:1); iii) Vero cells incubated with MHI 1761 supplemented with rNheC (ratios of NheB:NheC 1:1, 2.5:1, 5:1). Cell treatment was carried out at 37°C for 30 min. After washing the cells twice in 1% BSA-PBS primary antibodies were added at 2 µg ml^−1^ (MAb 1E11 for detection of NheB and unspecific MAb 5B2 for isotype control). MAbs were allowed to bind for 30 min, followed by washing twice and incubation with secondary Alexa Fluor 488-conjugated anti-mouse secondary antibody (Invitrogen, Germany). After two final rounds of washing, cells were analyzed using a FACSCalibur (BD Bioscience, USA) and CellQuestPro software (BD Bioscience, USA). Prior to analysis propidium iodide (2.5 µg ml^−1^) was added to the cell suspension.

### Sandwich Enzyme Immunoassay for Detection of NheC

Sandwich enzyme immunoassays (sandwich EIAs) were established in order to enable the sensitive detection of NheC in solution. After optimization of techniques as described previously [21], the following protocol was established: microtiter plates were coated with NheC-specific rabbit antiserum at a dilution of 1:1,000 in bicarbonate buffer (0.05 mol l^−1^, pH 9.6) over night at room temperature. After blocking of free protein binding sites with 3% sodium-caseinate-PBS for 30 min and a washing step, serial dilutions (in PBS containing 0.05% Tween 20) of purified rNheC or *B. cereus* supernatants were added for 1 h. The next washing step was followed by addition of the MAbs reactive with rNheC diluted in 1% sodium-caseinate-PBS (MAbs 1E12 and 2F10: 2.5 µg ml^−1^, MAbs 2G8 and 3D6: 1.25 µg ml^−1^) and another incubation for 1 h. Bound MAbs were detected by a rabbit anti-mouse antibody labeled with horseradish peroxidase (Dako, Germany; diluted 1:2,000 in 1% sodium-caseinate-PBS).

### Sandwich EIA for Detection of NheB-NheC Complexes

A sandwich EIA was used to detect NheB-NheC complexes by combining MAb 3D6 and MAb 1E11, specific for NheC and NheB, respectively. Microtiter plates were coated with MAb 3D6 (3 µg ml^−1^) diluted in PBS. Culture supernatants of *B. cereus* strains were added in serial dilutions. After a washing step, horseradish peroxidase-labeled MAb 1E11 was added at a dilution of 1:1,500 in 1% sodium-caseinate-PBS.

### Dot Blot Analyses

Purified rNheC (100 µl per dot) was applied to a PVDF-membrane (Immobilon-P; Millipore, USA) either as a dilution series ranging from 100 to 6 ng ml^−1^ or adjusted to a constant concentration of 25 ng ml^−1^. After removal from the dotting chamber, membranes were blocked with 3% sodium-caseinate-PBS overnight. NheB (from supernatant of MHI 1672) was diluted in PBS to concentrations between 250 and 2.5 ng ml^−1^, and overlaid on the dots for 1 h. After washing, membranes were incubated with NheB-specific MAb 1E11 (1 µg ml^−1 ^in 3% sodium-caseinate-PBS containing 0.025% Tween 20) for 1 h and horseradish-peroxidase-labeled rabbit anti-mouse secondary antibody (Dako, Germany; diluted 1:2,000 in 1% sodium-caseinate-PBS) for 1 h. After incubation with Super Signal West Femto Maximum Sensitivity Substrate (Pierce, USA) signals were recorded on a Kodak image station (Eastman Kodak, USA).

### Crosslinking of NheB and rNheC

Purified NheB and rNheC were incubated at molar ratios of 1:1, 1:2 and 1:10 for 30 min at 4°C. Crosslinking was achieved by addition of a 1000-fold molar excess of DSP (Dithiobis[succinimidylproprionate]; Thermo Scientific, Germany) for 5 min at room temperature. Crosslinking was stopped by the means of 0.1 M Tris base. Samples were subsequently prepared for and submitted to Western immunoblot analysis. NheC-specific MAb 3D6 served as detection antibody (3 µg ml^−1^ in 3% sodium-caseinate-PBS containing 0,025% Tween 20).

## Results

### Monoclonal Antibodies against NheC

#### Production

**Figure 1:**
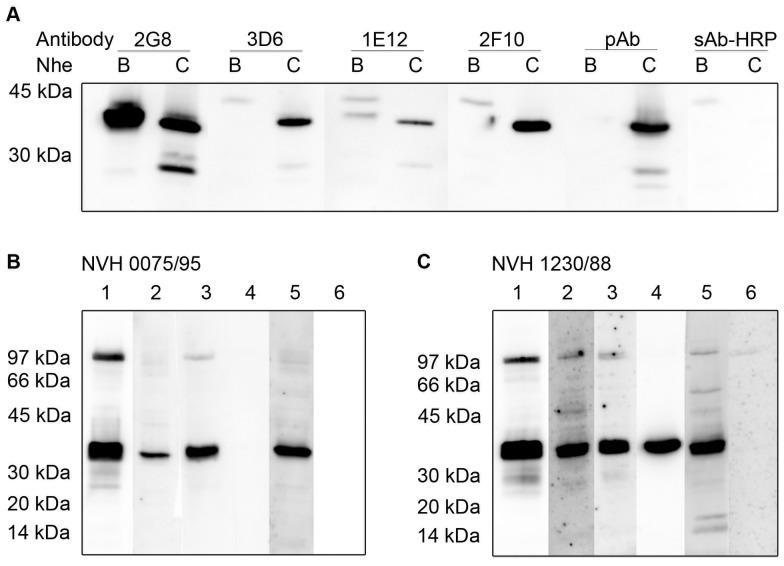
Western immunoblots showing reactivity of the monoclonal antibodies with purified Nhe-components and exoprotein preparations of *B.cereus* strains. (A) Reactivity with NheB and purified rNheC; rabbit antiserum (rAs) against NheC served as a positive control. Reactivity between 20 and 30 kDa represented protein degradation. Negative control, peroxidase-labeled secondary antibody (sAb-HRP) only, showed a minor band at approximately 43 kDa in each NheB lane due to unspecific reaction of secondary antibody. Reactivity of the antibodies with exoprotein preparations from *B. cereus* strain NVH 0075/95 (B) and *B. cereus* strain NVH 1230/88 (C), each concentrated 20-fold: MAb 2G8 (lane 1), MAb 3D6 (lane 2), MAb 1E12 (lane 3), MAb 2F10 (lane 4), rAs (lane 5) and the negative control (lane 6) as decribed in (A).

Since earlier attempts to produce NheC-specific MAbs by using crude toxin preparation failed, highly purified recombinant NheC was used for the immunization of mice. Immunoaffinity chromatography-purified fractions of rNheC contained 10 to 20 µg ml^−1^ of rNheC as determined by SYPRO Ruby protein staining of SDS-gels and showed no visible impurities (Fig. S1). NheC-specific rabbit antiserum [15] showed reactivity against purified rNheC, both in indirect EIA and in Western immunoblotting, revealing a band of approximately 37 kDa (Fig. 1A). Furthermore, after combining purified rNheC (1 ng ml^−1^) with cell free supernatant of *B. cereus* strain MHI 1672 (producing NheA and NheB) cytotoxic effects on Vero cells were induced. Purified rNheC was used for immunization and two booster injections of mice. A total of 19 hybridoma cell lines secreting antibodies reacting with rNheC were identified. For further characterization, three hybridomas producing IgG antibodies and one producing an IgM antibody were chosen (Table 1).

**Table 1:** Characteristics of the monoclonal antibodies.

Monoclonal antibody	Subtype	Specificity	Sensitivity (ng ml^−1^)^a^		Additivity index (%)^b^			
			rNheC	NheB	2G8	3D6	1E12	2F10
2G8	IgG_1_	NheB, C	10	49	−	81	38	83
3D6	IgM^c^	NheC	25	n.d.^d^		−	90	80
1E12	IgG_2a_	NheB, C	16	66			−	98
2F10	IgG_1_	NheC	65	n.d.				−

a Values given represent the concentration of the coating antigen, which resulted in an absorbance of 0.5 units under the conditions of the indirect EIA.

b A low additivity index (<50%) indicates competition of the respective MAbs for the same binding site. A high value indicates simultaneous binding to different epitopes [20].

c Monomer.

d Not detectable.

#### Properties

Western immunoblot and indirect EIA were performed to characterize the specificity of the four monoclonal antibodies (Table 2, Fig. 1). All four monoclonals showed immunoreactivity in EIA and revealed a distinct band at 37 kDa against rNheC (derived from NVH 1230/88) in Western immunoblotting (Table 2 and Fig. 1A). A band of same size was observed when applying crude culture supernatant of *B. cereus* strain NVH 1230/88 (Table 2 and Fig. 1C). Crude culture supernatant of *B. cereus* strain NVH 0075/95 yielded similar bands at 37 kDa for three of the MAbs, whereas MAb 2F10 showed no reactivity (Table 2 and Fig. 1B). In order to check potential cross-reactivity of the monoclonal antibodies with other Nhe-components, rNheA, purified NheB and a supernatant of *B. cereus* strain MHI 1672 (producing both NheA and NheB) were tested in the indirect EIA and the Western immunoblot assay. None of the four monoclonal antibodies bound to rNheA (Table 2). Two antibodies (MAb 2G8 and MAb 1E12), however, revealed a distinct reactivity with NheB (Table 2 and Fig. 1A). To further characterize the binding of the antibodies to NheC and NheB, indirect EIAs were carried out, using plates coated with serial dilutions of purified rNheC and NheB, respectively. The concentration of the coating antigen giving the same assay response (OD = 0.5) was used as a relative measure of the antibodieś binding strength allowing comparison of assay sensitivity (Table 1). All antibodies were able to detect rNheC in the low nanogram-range and reactivity of MAb 2G8 and MAb 1E12 with NheB was 4–5 fold lower compared to rNheC.

**Table 2:** Reactivity of Nhe-components and *B. cereus* strains with the monoclonal antibodies.

	Monoclonal antibody^a^			
Component/Strain	2G8	3D6	1E12	2F10
rNheC^b^	+	+	+	+
NheB^c^	+	−	+	−
RNheA	−	−	−	−
NVH 1230/88	+	+	+	+
MHI 1672	+	−	+	−
NVH 0075/95	+	+	+	−

a Positive (+) and negative (−) results in the indirect EIA.

b Purified recombinant NheC, originating from *B. cereus* strain NVH 1230/88.

c Purified NheB, from *B. cereus* strain MHI 1672 (producing NheA and NheB).

Neutralization capacity of the monoclonal antibodies was tested on Vero cells under different experimental conditions as described earlier [17]. Neither in the simultaneous nor the consecutive assay setup neutralization of Nhe-related cytotoxic activity was observed.

#### Epitopes

**Figure 2:**
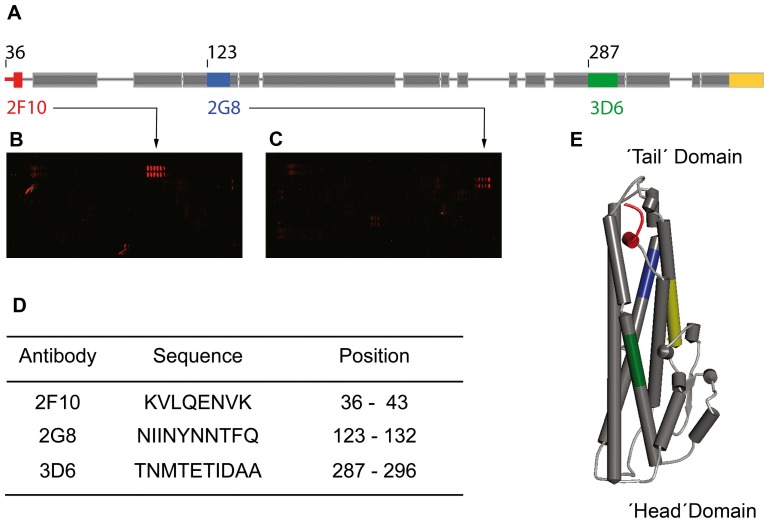
Epitopes of MAb 2G8, 3D6 and 2F10 within the sequence of NheC (derived from *B. cereus* strain NVH 1230/88, GenBank accession code: CAB53340.2). (A) Localisation of the epitopes within the secondary structure model: MAb 2F10 (red), MAb 2G8 (blue) and MAb 3D6 (green); the yellow area depicts the peptide used for production of the rabbit antiserum; amino acids 1 to 35 are not shown. (B) and (C) Epitope mapping on a peptide microarray, exemplified illustrations for MAb 2F10 (B) and MAb 2G8 (C); red double spots indicate the peptide consensus motif, irregular spots are impurities. (D) Sequences and positions of the mapped epitopes. (E) Homology model of NheC obtained by SWISS-MODEL [33]–[35], using the Hbl-B crystal structure (PDB ID:2nrj) [12] as a template, showing the MAb epitopes colored according to (A) (drawn by Accelrys Discovery Studio 3.0 Visualizer).

MAb 2F10 showed a unique reactivity pattern both in EIA and in Western immunoblot analyses. Epitope-mapping revealed a well-defined epitope close to the N-terminus of rNheC (Fig. 2). Clustal alignment of published *nheC* sequences indicated that this region of *nheC* frequently shows heterology resulting in amino acid exchanges. Thus the distinct reactivity pattern of MAb 2F10 (Fig. 1), i.e. clear reactivity with rNheC and *B. cereus* strain NVH 1230/88 (used for generation of rNheC), but no reactivity with *B. cereus* strain NVH 0075/95, reflects just this local sequence dissimilarity between both strains. Comparison of the amino acid sequences of NVH 1230/88 and NVH 0075/95 (Fig. S2) reveals an exchange of amino acids at position 37 and 38 as well as at positions 42 to 43. Thus the epitope of MAb 2F10 (KVLQENVK) is replaced by the sequence KIQQENAN in strain NVH 0075/95. This difference is enough to fully prevent antibody binding, meaning that assays using MAb 2F10 will selectively detect *B. cereus* strains matching the NVH 1230/88 sequence of the antibody binding site.

MAb 2G8 showed not only a distinct band towards recombinant NheC at 37 kDa, but also an additional band at 39 kDa indicating cross-reactivity with NheB (Table 2, Fig. 1). The strong reactivity with the top band at approximately 100 kDa is common for antibodies reacting with NheB [15], and the band was identified to represent an oligomer of NheB by two-dimensional gel electrophoresis and N-terminal sequencing [22]. Cross-reactivity of some of the antibodies with NheB was expected, because substantial sequence homologies exist between the B- and C-component of the Nhe-complex [9]. The epitope of MAb 2G8 (Fig. 2), as detected by peptide microarray, covers amino acids NIINYNNTFQ, a sequence that is sufficiently (NIINYN) conserved in NheB at position 131 to 136 to allow antibody binding (Fig. S2). According to the structural model of NheC (Fig. 2E), this epitope is located within a predicted α-helix. Considering the consistent reactivity pattern of MAb 2G8 found in both EIA (Table 1 and Table 2) and Western immunoblot analyses (Fig. 1A), a continuous binding site can be assumed.

According to the results of the peptide microarray, the most likely epitope of MAb 3D6 was in the range of amino acids 287 to 296 (TNMTETIDAA). MAb 3D6 was an IgM-monomer, highly specific for NheC, and its binding site was within a predicted α-helix close to the C-terminal end (Fig. 2). Reactivity with NheC in EIA and Western immunoblot was consistent (Table 2, Fig. 1), indicating a continuous epitope, which seemed to be conserved in all *B. cereus* strains tested.

MAb 1E12 showed a more polyclonal reactivity pattern and a putative epitope was located within amino acids 116 to 140 (QIMKTDQNIINYNNTFQSYYNDMLI), which includes the sequence NIINYNNTFQ recognized by MAb 2G8. In the Western immunoblot MAb 1E12 also showed an additional band at 39 kDa indicating cross-reactivity with NheB (Table 2, Fig. 1). These results indicated a main binding region similar to that of MAb 2G8, which was in accordance with the low additivity index obtained for this antibody pair (Table 1). Competitive binding due to overlapping or sterically close epitopes was, however, not found for any of the other possible combinations of antibodies (Table 1). Furthermore, MAb 1E12 showed a much lower reactivity in the Western immunoblot than MAb 2G8 (Fig. 1A), but only slightly less sensitivity in EIA analyses (Table 1). Also the cross-reactivity pattern with NheB was not consistent in both methods. Most likely, this could be due to binding to a discontinuous epitope, in which the binding site is completed by amino acids adjacent in the tertiary structure, but not after SDS-PAGE.

#### Immunocytochemistry

**Figure 3:**
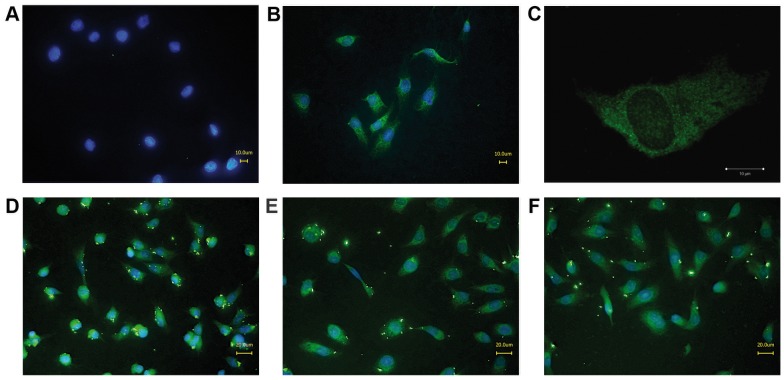
Monoclonal antibodies react with cell-bound rNheC. Vero cells, untreated (A) and treated with 400 ng ml^−1^ rNheC were stained with MAb 3D6 (B). A single cell image obtained by confocal laser scanning microscopy is shown in (C). Vero cells treated with 1.6 µg ml^−1^ rNheC and stained with MAb 2G8 (D), MAb 1E12 (E) as well as MAb 2F10 (F). All MAbs were detected by Alexa Fluor 488-labeled secondary anti-mouse antibody.

Immunofluorescence pictures showed that each of the monoclonal antibodies was able to bind to cell-bound rNheC (Fig. 3). Particularly MAb 3D6 and a low concentration of rNheC (0.4 µg ml^−1^) gave a homogeneously distributed staining using conventional fluorescence microscopy (Fig. 3B) as well as confocal laser scanning imaging (Fig. 3C). At higher concentrations (1.6 µg ml^−1^ rNheC), the antibodies tended to produce a more punctuated fluorescence pattern (Fig. 3D–F), probably due to formation of multimeres of rNheC. Overall, these results indicated that the epitopes of the monoclonal antibodies were not involved in cell binding of NheC.

### Complex Formation between NheB and NheC

#### Only trace amounts of NheC are detectable in culture supernatants of *B. cereus*

**Figure 4:**
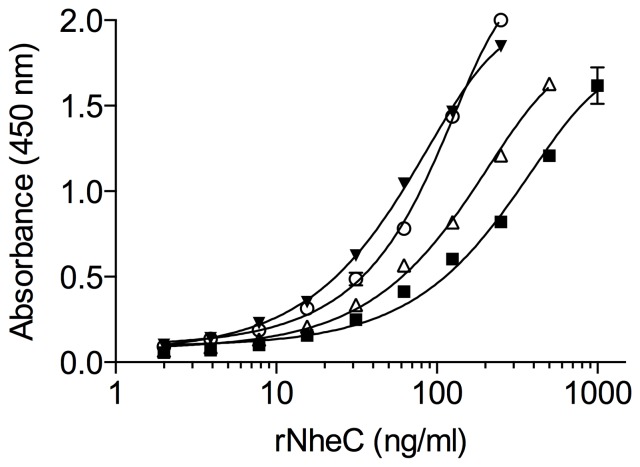
Sandwich EIA for the detection of NheC. Rabbit antiserum raised against NheC was used for coating the microtiter plates (capture antibodies) and the monoclonal antibodies for detection (MAb 3D6, closed triangle; MAb 2G8, open circle; MAb 1E12, open triangle; MAb 2F10, closed square). Three independent experiments were performed for each combination and a representative curve is shown.

To examine the presence of NheC in *B. cereus* culture supernatants, sandwich EIAs were established and validated by using rNheC. Using the previously described polyclonal rabbit antiserum against NheC [15] for capture and the newly developed MAbs for detection, as low as 2–10 ng ml^−1^ of NheC could be detected (Fig. 4). The most intense signal was obtained by using MAb 3D6, the linear range of the dose-response curve was between 20 and 200 ng rNheC per ml. Using this sensitive assay, the NheC productivity of *B. cereus* strains was analyzed. However, only trace amounts of NheC were found in the respective culture supernatants, detectable concentrations ranged from <0.01 to 0.03 µg ml^−1^ (Table 3). Considering that the NheB concentrations found in the supernatants were mostly above 1 µg ml^−1^, and since a ratio between NheB and NheC of approximately 10:1 is necessary for maximum toxicity [10], [14], the corresponding NheC levels were expected to be above 0.1 µg ml^−1^. Therefore, for most strains the NheC concentration measured, represented less than 1% of the expected values.

**Table 3:** Concentrations of NheB and NheC compared to the titer of NheB-C complexes in supernatants of *B. cereus* strains.

Strain	NheB(µg ml^−1^)^a^	NheC(µg ml^−1^)^b^	NheB-Ccomplex^c^
Reference strains			
NVH 0075/95	11.50	<0.01	+ +
NVH 1230/88	5.81	0.08	+ +
NVH 0391/98	<0.01	<0.01	−
Food related strains			
MHI 1477	4.64	<0.01	+ +
MHI 1493	13.97	<0.01	+ +
MHI 1496	5.43	<0.01	+ +
MHI 1503	12.41	<0.01	+ +
MHI 1507	5.03	<0.01	+
MHI 1522	4.34	0.02	+ +
MHI 1527	4.19	0.01	+ +
MHI 1541	2.22	0.03	+ +
MHI 1543	4.44	0.01	+ +
MHI 1556	<0.01	<0.01	−
MHI 1668	1.27	0.01	+
MHI 1670	1.15	0.03	+ +
MHI 1676	<0.01	<0.01	−
MHI 1692	4.16	0.01	+ +
MHI 2963	5.14	0.01	+ +
MHI 2964	5.54	<0.01	+ +
MHI 2965	5.49	<0.01	+ +
MHI 2967	4.50	<0.01	+
MHI 2968	4.90	<0.01	+ +
MHI 2970	0.10	<0.01	+ +
MHI 2971	6.31	<0.01	+
MHI 2972	9.76	<0.01	+ +

a Sandwich EIA according to [21].

b Sandwich EIA described in this study.

c Sandwich EIA described in this study, results expressed as reciprocal values of the titers, classified as follows: −, titers of <3; +, titers of 3 to 99; ++, titers ≥100.

#### NheC forms a complex with NheB in natural culture supernatants of *B. cereus*

**Figure 5:**
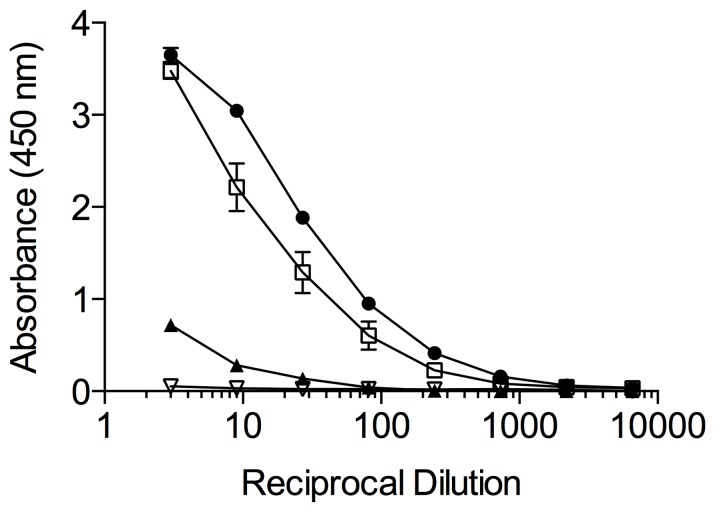
Sandwich EIA for detection of NheB-NheC complexes. Dilution curves obtained for the reference strains NVH 0075/95 (open squares) and NVH 1230/88 (closed circles) in the sandwich EIA (capture antibody MAb 3D6 against NheC; detection antibody MAb 1E11-HRP against NheB). Purified NheB (closed triangles) and rNheC (open triangles) served as negative controls. Three independent experiments were performed for each strain and a representative curve is shown.

To test the hypothesis that NheC is bound to NheB in culture supernatants, a sandwich EIA was designed enabling the specific detection of these complexes. Here, the NheC-specific MAb 3D6 served as capture antibody and detection of bound complexes was enabled by the use of the previously described NheB-specific MAb 1E11 [15]. The assay was optimized using the Nhe positive reference strains NVH 1230/88 and NVH 0075/95. *B. cytotoxicus* NVH 0391/98, producing highly divergent Nhe proteins [23], which did not react with the MAbs against NheB and NheC, served as a negative control. Analyzing serial dilutions of cell-free culture supernatants of these strains proved the applicability of the sandwich EIA and revealed that NheC forms stable complexes with NheB under these growth conditions (Fig. 5). The reference strains NVH 0075/95 and NVH 1230/88 showed a clear positive reaction while NVH 0391/98 was negative. Expanding the analyses to 22 other *B. cereus* strains underlined these results. NheB negative strains MHI 1556 and MHI 1676 showed no reactivity in the complex-specific EIA, whereas in the supernatants of all other strains (producing NheB and NheC) immunoreactive complexes were detected (Table 3).

#### Concentration of the complex between NheB and NheC is inversely correlated with toxicity

**Figure 6:**
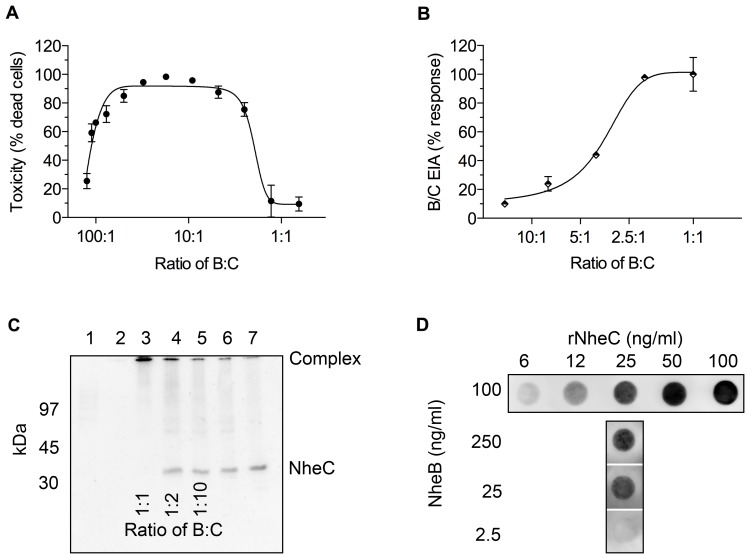
Comparison of toxicity and complex formation. (A) The bell-shaped dose-response curve obtained in the WST-assay showed maximum cytotoxicity between a 50:1 and 5:1 ratio of NheB and NheC. (B) Increase of complex formation between NheB and NheC as detected by sandwich EIA, reaching maximum assay response above a 2.5:1 ratio. (C) Crosslinking analysis of the oligomerization of NheB and NheC. Purified NheB and rNheC were mixed at molar ratios of 1:1 (lane 3), 1:2 (lane 4) and 1:10 (lane 5), crosslinked and detected by NheC-specific MAb 3D6. Purified NheB (lane 1) and rNheC (lane 7) as well as NheB mixed with DSP (lane 2) and rNheC mixed with DSP (lane 6) served as controls. (D) Dot blot analysis of complex formation between NheB and NheC. The concentration of rNheC used for membrane coating is indicated on the horizontal axis, the concentration of NheB applied in the second step is shown on the vertical axis. Three to four independent experiments were performed for each combination and a representative blot is shown.

To address the question whether these complexes are important for cytotoxic action of Nhe, we compared the level of complex formation with cytotoxicity on Vero cells (Fig. 6). A diluted supernatant of strain MHI 1672, containing approximately 40 ng ml^−1^ each of NheA and NheB, was supplemented with rNheC. At a 1:1 ratio between NheB and NheC, toxicity was reduced to background level. The cytotoxicity reached a maximum plateau at ratios between 5:1 and 50:1. A further decrease of the NheC concentration to or below a 100:1 ratio abolished cytotoxicity (Fig. 6A). The level of complex formation between NheB and NheC was directly correlated with the relative concentrations of NheB and NheC (Fig. 6B). To further prove the result obtained in the immunoassay, purified NheB and rNheC were mixed at different ratios, cross-linked with DSP and the resulting products were analyzed by SDS-PAGE and Western immunoblotting using MAb 3D6 (Fig. 6C). Without NheB and at excess concentrations of rNheC the monomeric form of NheC was detectable, while it gradually disappeared with increasing concentrations of NheB. At a 1:1 ratio between NheB and NheC the NheC monomer was no longer detectable and the band corresponding to the covalently linked complex became most intense. In addition, dot blots using MAb 1E11 against NheB clearly showed binding of NheB to membranes coated with rNheC (Fig. 6D). The binding of NheB was dependent on the concentration of rNheC on the dots, and maximum binding was achieved at a 1:1 ratio between NheB and NheC or at excess concentrations of NheB.

### Complex Formation between NheB and NheC Enhances and Inhibits Cell Binding of NheB

**Figure 7:**
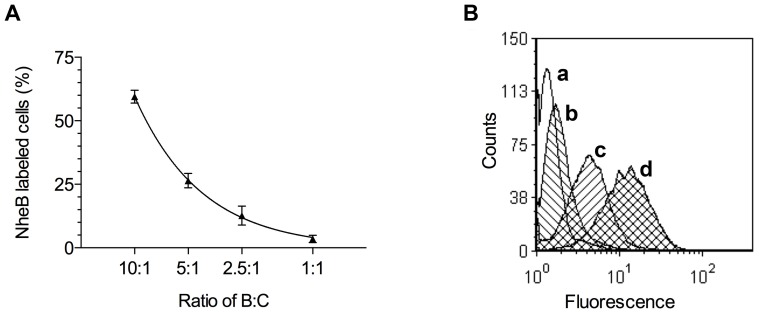
Binding of NheB to Vero cells. (A) Decrease of NheB binding to Vero cells as determined by flow cytometry. (B) Histogram showing the overlay of NheB-specific fluorescence counts (FL1-H) for the isotype control (a), a 1:1 (b), 5:1 (c), and 10:1 (d) ratio between NheB and NheC.

In order to quantify the percentage of cell-bound NheB-C complexes, Vero cells were examined by flow cytometry (Fig. 7A, B). Cells were incubated with culture supernatant from MHI 1761 (producing NheB and NheC at a 10:1 ratio) with and without supplementation of rNheC. The supernatant was additionally analyzed by sandwich EIA for quantification of NheB as well as by the complex-specific sandwich EIA. Accordingly supernatants of the strains were diluted to a final concentration of app. 40 ng ml^−1^ NheB. Further experimental set-ups included i) no supplementation of rNheC, ii) supplementation of rNheC to achieve ratios between NheB and NheC of 1:1, 2.5:1 and 5:1. As the tripartite Nhe toxin is known to induce pore-formation on the target cells, all samples were assayed in the presence of propidium iodide (PI). Although the different treatments did not affect cell morphology and granulation as determined by forward scatter and side scatter signals, incubation with diluted MHI 1761 resulted in 5–10% cells positive for PI fluorescence. Effects caused by the supplementation of additional rNheC were analyzed in the PI negative cells only. The diluted MHI 1761 culture supernatant with a natural ratio between NheB and NheC (10:1) resulted in 63% ±5.22 cells positive for NheB binding. Adjusting NheB:NheC ratios to 5:1 and 2.5:1 by addition of rNheC, reduced the NheB signal to 26% ±2.44 and 13% ±3.04, respectively. Finally, a ratio between NheB and NheC of 1:1 gave a positive signal in only 4% ±1.24 of the cells.

## Discussion

Besides Hbl, Nhe represents a second tripartite enterotoxin involved in the etiology of *B. cereus* food poisoning [7]. The *nhe* genes could be found in most *B. cereus* isolates [24]–[27], and Nhe seems to be the most prevalent enterotoxin produced by *B. cereus*. Recent studies on the mode of action of Nhe [14], [17] indicated that NheC is able to bind to NheB in solution and that both components can bind to cell membranes. Additionally, it was shown that the Nhe components require a specific binding order and NheC was mandatory in the priming step to achieve maximum cytotoxicity. Further in depth studies on the role of the interaction between NheB and NheC in Nhe-induced cytotoxicity were, however, hampered by the lack of powerful analytical tools such as monoclonal antibodies against the C-component.

For this purpose we established four cell lines secreting antibodies reactive towards rNheC. Two of the MAbs (2G8 and 1E12) showed cross-reactivity with NheB, but MAbs 2F10 and 3D6 were highly specific for NheC and particularly suited for the intended studies. Recently we showed that inhibition of ordered binding of the individual Nhe components, particularly by preventing binding of NheA to NheB on the cell surface by MAb 1E11 against NheB, was an efficient way to neutralize Nhe toxicity [17]. However, none of the MAbs against NheC developed in this study, reduced Nhe toxicity, not even when NheC was pre-incubated with MAbs prior to addition of the other components. This result indicates that the epitopes of the MAbs against NheC are not involved in the interaction between the individual Nhe components and probably do not interfere with cell binding of NheC. The latter assumption was proved by fluorescence microscopic images, which clearly demonstrated a NheC-dependent staining of Vero cells in presence of the MAbs (Fig. 3). These results are in agreement with the structural model of NheC (Fig. 2E), from which it could be predicted that the epitopes of the antibodies are not located in the head region and show sufficient distance to the putative trans-membrane region containing the beta-hairpin [14] in order to not inhibit binding of NheC to the cell surface.

According to the current model, the first step in the mode of action of Nhe is binding of NheB and NheC to the cell surface, but interaction between both components occurs most likely before cell binding. Since the hetero-oligomers of NheB and NheC formed in solution are not stable under SDS-PAGE conditions (Fig. S3), evidence for complex formation has been provided by immunoblotting experiments on native gels [10], as well as by co-immunoprecipitation of NheB and NheC [14]. Here we further confirm the formation of hetero-oligomers between NheB and NheC by crosslinking analyses and dot blotting. However, a major drawback of all these methods is that they only can demonstrate complex formation in artificial systems requiring purified Nhe components. Therefore, we wanted to establish a simple method for the detection of complexes between NheB and NheC, applicable to natural culture supernatants. For this purpose, we tested the suitability of the antibodies to detect complexes between NheB and NheC in a sandwich immunoassay. In combination with the previously developed rabbit antiserum against NheC, all four MAbs were able to detect rNheC in a sandwich immunoassay (Fig. 4) with a detection limit below 10 ng ml^−1^. When however, culture supernatants were analyzed for NheC, no or only minor amounts were detected (Table 3, data shown for MAb 3D6). The lack of detectable NheC indicated that either the concentration of NheC was lower than expected or that at least one binding site of the antibodies was not accessible in the culture supernatants due to complex formation between NheC and NheB. To test the latter hypothesis, we constructed a sandwich immunoassay using the NheC-specific monoclonal antibody 3D6 for capture and MAb 1E11 [15] against NheB (labeled with HRP) for detection. As expected, this assay gave a positive result for the tested supernatants, indicating the presence of NheB-NheC complexes. Considering the low amount of NheC detected in the NheC-specific assay, it could be concluded that in culture supernatants more than 90% of NheC is bound to NheB. On the other hand, there was no correlation between the concentration of NheB and the titer observed in the NheC/B assay in culture supernatants (Table 3). Though all three Nhe components are transcribed from one operon after activation by the global transcriptional activator PlcR [28], it is not fully understood which mechanisms lead to the different secretion levels of NheB and NheC. A recent study, showing that the premature Nhe proteins contain Sec-type signal peptides and are secreted via the Sec pathway, did not indicate accumulation of individual premature components in the cell [29]. On the other hand, a stem-loop structure between *nheB* and *nheC* was predicted from the *nhe* sequence [9] and it has been suggested that the relative low level of expression of NheC is caused by translational repression due to secondary structure formation at or close to the ribosome binding site [10]. As the intergenic sequences between *nheB* and *nheC* are variable (unpublished data), it could be assumed that the extent of this repression differs from strain to strain, leading to variable concentration ratios between NheC and NheB, finally resulting in different levels of complex formation.

To address the question about the function of the complexes between NheB and NheC, we compared complex formation to cytotoxicity on Vero cells (Fig. 6A, B). Cell binding of NheB was quantified by flow cytometry (Fig. 7A, B). The relative amount of complexes was directly correlated with the ratio between NheB and NheC. Toxicity, however, showed a bell-shaped dose-response curve with a maximum level between NheB:NheC ratios of 50:1 and 5:1. Most interestingly, flow cytometry showed that the highest percentage of NheB binding to Vero cells was found when the ratio between NheB and NheC was in the above-mentioned range. Significantly less NheB positive cells were seen when the ratio between NheB and NheC was lower than 5:1, accompanied by high levels of complex formation. It has been speculated that the mechanism behind the inhibitory effect of excess NheC is the formation of NheB-NheC complexes, which are substantially impaired in cell binding [14]. The results shown in this study indicate a more complex situation. A defined level of NheB-NheC complexes as well as a sufficient amount of free NheB seem to be necessary for efficient cell binding and toxicity of Nhe. Interestingly, we have shown previously that NheB as well as NheC were able to bind to Vero cells individually, however, only cells primed with NheC could be lysed by the addition of the respective other two components after a washing step [14]. In the light of the data presented here, it must be concluded that formation of functional complexes between NheB and NheC is still possible after cell binding of NheC but not after cell binding of NheB. A possible explanation is that at the relatively high concentrations used in the former study, NheB is ´unspecificallý attached to the cell surface and no longer available for any further action. The activity of NheC, on the other hand, seems not to be influenced by cell binding and NheB, added in the second step, will be able to bind to cell bound NheC. From the experimental set-up it can, however, not be excluded that NheC is released from the cell surface during the second incubation step and that the NheB-NheC complexes are formed in solution.

According to the current model, the first step in the mode of action of Nhe is associated with binding of NheC and NheB to the cell surface [14]. The data presented here allow a more detailed view on this first step and we propose the following refined model. Assuming that the three Nhe components are secreted individually using the Sec pathway, the first step outside the bacterial cell will be the formation of stable complexes between NheB and NheC. The NheB-NheC complexes will then bind to the cell surface, probably to a specific surface structure recognizing the NheB-NheC complex as well as free NheC, but not free NheB. This step will be accompanied by conformational changes [30], which allow subsequent attachment of free NheB but not of NheB-NheC complexes. Binding and oligomerization of a sufficient amount of free NheB are necessary to form a ring-shaped structure. Following this step, NheA will be able to bind to cell-bound NheB and complete the trans-membrane pore.

Nhe belongs to the α-helical pore-forming toxins with structural similarity to ClyA [11]. In general, pore-forming toxins are secreted as soluble proteins, which bind to the target cell, oligomerize on the cell membrane and finally form a transmembrane channel [31], [32]. Among pore-forming toxins, Nhe is one of the very few known toxin complexes requiring three different proteins for cytotoxic action. Here we describe another fascinating aspect of Nhe, namely the complex formation between two of its components in solution before cell binding. The specific interaction between NheB and NheC represents an important step during the complex mode of action of Nhe and elucidation of the structural and biochemical properties of this protein-protein interaction will be an essential step to provide an overall model explaining pore formation by Nhe.

## Supporting Information

Figure S1 **SYPRO Ruby stained SDS-PAGE showing purified rNheC.** Lane 1 and 2, IAC flow-through of two independent lysates; lane 3 and 4, eluted rNheC at 37 kDa; lane 3 is corresponding to flow-through of lane 1, lane 4 is related to lane 2; lane 5, BSA at 66 kDa (adjusted to 5 µg ml^−1^) as a standard for quantification.
(PDF)

Figure S2 **Clustal alignment of NheC (derived from** ***B. cereus*** **strains NVH 1230/88 and NVH 0075/95) and NheB (derived from** ***B. cereus*** **strains NVH 1230/88 and NVH 0075/95).**
(PDF)

Figure S3 **Western immunoblot showing lability of B-C-oligomers under SDS-PAGE conditions.** Lanes 1 and 3, purified rNheC and NheB mixed at a molar ratio of 1:1; lanes 2 and 4, purified rNheC as a control. Lanes 1 and 2, detection by NheB-specific MAb 1E11 (NheB at 39 kDa). Lanes 3 and 4, detection by NheC-specific MAb 3D6 (NheC at 37 kDa). Lanes 5 and 6, crosslinking of NheB and rNheC mixed at a molar ratio of 1:1 (lane 5) and 1:2 (lane 6); detection by NheC-specific MAb 3D6; crosslinked B-C-complexes at the top of lanes 5 and 6.
(PDF)
