# Supplementary material for: Complex Formation between NheB and NheC Is Necessary to Induce Cytotoxic Activity by the Three-Component Bacillus cereus Nhe Enterotoxin
Source: PLoS One. 2013 Apr 30;8(4):e63104. doi: 10.1371/journal.pone.0063104 (PMC3639968; doi:10.1371/journal.pone.0063104)
Supplement: Figure S1 — SYPRO Ruby stained SDS-PAGE showing purified rNheC. Lane 1 and 2, IAC flow-through of two independent lysates; lane 3 and 4, eluted rNheC at 37 kDa; lane 3 is corresponding to flow-through of lane 1, lane 4 is related to lane 2; lane 5, BSA at 66 kDa (adjusted to 5 µg ml−1) as a standard for quantification. (PDF) [file pone.0063104.s001.pdf]

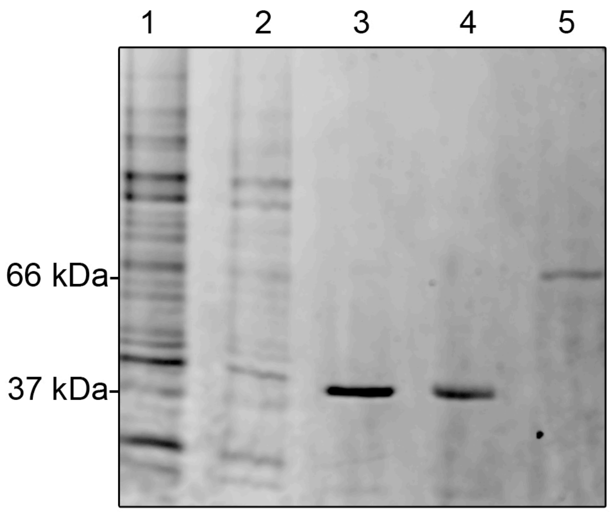

Figure S1. SYPRO Ruby stained SDS-PAGE showing purified rNheC. Lane 1 and 2, IAC flow-through of two independent lysates; lane 3 and 4, eluted rNheC at 37 kDa; lane 3 is corresponding to flow-through of lane 1, lane 4 is related to lane 2; lane 5, BSA at 66 kDa (adjusted to 5 µg ml^{-1}) as a standard for quantification.
