# Supplementary material for: Complex Formation between NheB and NheC Is Necessary to Induce Cytotoxic Activity by the Three-Component Bacillus cereus Nhe Enterotoxin
Source: PLoS One. 2013 Apr 30;8(4):e63104. doi: 10.1371/journal.pone.0063104 (PMC3639968; doi:10.1371/journal.pone.0063104)
Supplement: Figure S2 — Clustal alignment of NheC (derived from B. cereus strains NVH 1230/88 and NVH 0075/95) and NheB (derived from B. cereus strains NVH 1230/88 and NVH 0075/95). (PDF) [file pone.0063104.s002.pdf]

```
NheC1)          MQKRFYKKCLLAVMIAGVATSNAFPLHPFAAEQNVKVL--------QENV   42
NheC2)          MQKRFYKKCLLTLMIAGVATSNAFPLHTFAAEQNVKIQ--------QENA   42
NheB3)          MTKKPYKVMALSALMAVFAAGNIMPAHTYAAESTVKQAPVHAVAKAYNDY   50
NheB4)          MTKKPYKVMALSALMAVFTAGNIMPAHTYAAESTVKQAPVHAVAKAYNDY   50
                * *: **   *: ::* .::.* :* *.:***..**           ::

NheC            KNYSLGPAGFQDVMAQTTSSIFAMDSYAKLIQNQQETDLSKISSINSEFK   92
NheC            NDYSLGPAGFQDVMAQTTSSIFAMDSYAKLIQNQQETDLSKISSINGELK   92
NheB            EEYSLGPEGLKDAMERTGSNALVMDLYALTIIKQGNVNFGNVSSVDAALK   100
NheB            EEYSLGPEGLKDAMERTGSNALVMDLYALTIIKQGNVNFGNVSSVDAALK   100
                ::***** *::*.* :* *. :.** **  * :* :.::.::**::. :*

NheC            GNMIQHQRDAKINAAYWLNNMKPQIMKTDQNIINYNNTFQSYYNDMLIAI   142
NheC            GNMIQHQRDAKMNAAYWLNSMKPQIMKTDQNIINYNNTFQSYYNDMLIAI   142
NheB            GKVIQHQDTARGNAKQWLDVLKPQLISTNQNIINYNTKFQNYYDTLVAAV   150
NheB            GKVIQHQDTARGNAKQWLDVLKPQLISTNQNIINYNTKFQNYYDTLVAAV   150
                *::****  *: **  **: :***::.*:*******..**.**: :: *:

NheC            DQKDSGKLKADLEKLYADIVKNQNEVDGLLGNLKSFRDRMAKDTNSFKED   192
NheC            DQKDSGKLKADLEKLYADIVKNQNEVDGLLGNLKAFRDRMAKDTNSFKED   192
NheB            DAKDKATLTKGLTRLSSSINENKAQVDQLVEDLKKFRNKMTSDTQNFKGD   200
NheB            DAKDKATLTKGLTRLSSSINENKAQVDQLVEDLKKFRNKMTSDTQNFKGD   200
                * **...*. .* :* :.* :*: :** *: :** **::*:.**:.** *

NheC            TNQLTAILASTNAGIPALEQQINTYNDSIKKSNDMVI-------------   229
NheC            TNQLTAILASTNAGIPALEQQINTYNDSIKKSNDMVI-------------   229
NheB            ANQITSILASQDAGIPLLQNQITTYNEAISKYNAIIIGSSVATALGPIAI   250
NheB            ANQITSILASQDAGIPLLQNQITTYNEAISKYNAIIIGSSVATALGPIAI   250
                :**:*:**** :**** *::**.***::*.* * ::*

NheC            ----------AGGVLCVALIT---CLAGG--PMIAVAKKDIANAEGEIAN   264
NheC            ----------AGGVLCVALIT---CLAGG--PMIAVAKKDIANAEREIAN   264
NheB            IGGAVVIATGAGTPLGVALIAGGAAAVGGGTAGIVLAKKELDNAQAEIQK   300
NheB            IGGAVVIATGAGTPLGVALIAGGAAAVGGGTAGIVLAKKELDNAQAEIQK   300
                          **  * ****:   . .**  . *.:***:: **: ** :

NheC            LKDRISGAQAEVVILTDVKNKTTNMTETIDAAITALQNISNQWYTVGAKY   314
NheC            LKDRISGAQAEVAILTDVKNKTTNMTETIDAAITALQNISNQWYTVGAKY   314
NheB            ITGQVTTAQLEVAGLTNIKTQTEYLTNTIDTAITALQNISNQWYTMGSKY   350
NheB            ITGQVTTAQLEVAGLTNIKTQTEYLTNTIDTAITALQNISNQWYTMGSKY   350
                :..::: ** **. **::*.:*  :*:***:**************:*:**

NheC            NNLLQNVKGITPEEFTFIKEDLHTAKDSWKDVKDYTEKLHEGVAK------- 359
NheC            NNLLQNVKGITPEEFTFIKEDLHTAKDSWKDVKDYTEKLHEGVAK------- 359
NheB            NSLLQNVDSISPNDLVFIKEDLNIAKDSWKNIKDYAEKIYAEDIKVVDTKKA 402
NheB            NSLLQNVDSISPNDLVFIKEDLNIAKDSWKNIKDYAEKIYAEDIKVVDTKKA 402
                *.*****..*:*:::.******: ******::***:**::    *
```

---

1) GenBank accession code: CAB53340.2 (NVH 1230/88)
2) GenBank accession code: AAZ82473.1 (NVH 0075/95)
3) GenBank accession code: CAB53339.2 (NVH 1230/88)
4) GenBank accession code: AAZ82472.1 (NVH 0075/95)

Figure S2. Clustal alignment of NheC (derived from *B. cereus* strains NVH 1230/88 and NVH 0075/95) and NheB (derived from *B. cereus* strains NVH 1230/88 and NVH 0075/95).
