# Supplementary material for: Complex Formation between NheB and NheC Is Necessary to Induce Cytotoxic Activity by the Three-Component Bacillus cereus Nhe Enterotoxin
Source: PLoS One. 2013 Apr 30;8(4):e63104. doi: 10.1371/journal.pone.0063104 (PMC3639968; doi:10.1371/journal.pone.0063104)
Supplement: Figure S3 — Western immunoblot showing lability of B-C-oligomers under SDS-PAGE conditions. Lanes 1 and 3, purified rNheC and NheB mixed at a molar ratio of 1:1; lanes 2 and 4, purified rNheC as a control. Lanes 1 and 2, detection by NheB-specific MAb 1E11 (NheB at 39 kDa). Lanes 3 and 4, detection by NheC-specific MAb 3D6 (NheC at 37 kDa). Lanes 5 and 6, crosslinking of NheB and rNheC mixed at a molar ratio of 1:1 (lane 5) and 1:2 (lane 6); detection by NheC-specific MAb 3D6; crosslinked B-C-complexes at the top of lanes 5 and 6. (PDF) [file pone.0063104.s003.pdf]

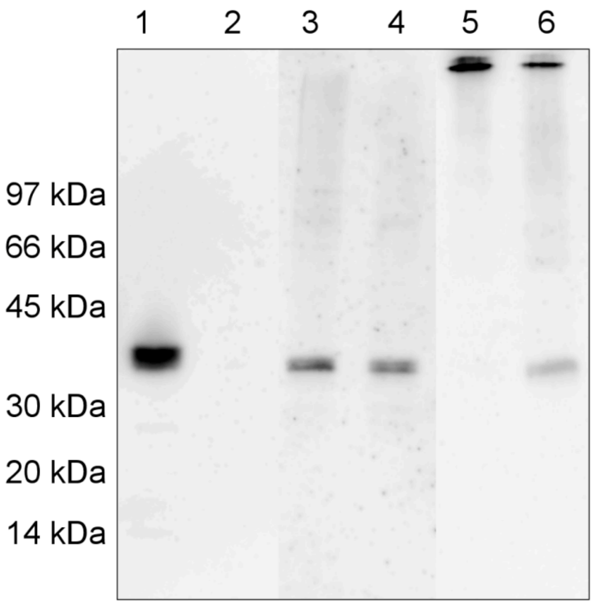

Figure S3. Western immunoblot showing lability of B-C-oligomers under SDS-PAGE conditions. Lanes 1 and 3, purified rNheC and NheB mixed at a molar ratio of 1:1; lanes 2 and 4, purified rNheC as a control. Lanes 1 and 2, detection by NheB-specific MAb 1E11 (NheB at 39 kDa). Lanes 3 and 4, detection by NheC-specific MAb 3D6 (NheC at 37 kDa). Lanes 5 and 6, crosslinking of NheB and rNheC mixed at a molar ratio of 1:1 (lane 5) and 1:2 (lane 6); detection by NheC-specific MAb 3D6; crosslinked B-C-complexes at the top of lanes 5 and 6.
